# Analysis of plasmon modes in Bi_2_Se_3_/graphene heterostructures via electron energy loss spectroscopy

**DOI:** 10.1038/s41598-024-81488-7

**Published:** 2024-12-28

**Authors:** Timothy Moorsom, Mairi McCauley, Ahmad Nizamuddin Bin Muhammad Mustafa, Sami Ramadan, Joel Burton, Satoshi Sasaki, Donald A. MacLaren, Peter K. Petrov

**Affiliations:** 1 School of Chemical and Process Engineering, University of Leeds, Leeds, LS2 9JT UK; 2 SUPA, School of Physics and Astronomy, University of Glasgow, Glasgow, G12 8QQ UK; 3 Department of Materials, Imperial College London, London, SW7 2AZ UK; 4 School of Physics and Astronomy, University of Leeds, Leeds, LS2 9JT UK; 5 FTKEK, Universiti Teknikal Malaysia Melaka, Malacca, Malaysia

**Keywords:** Surfaces, interfaces and thin films, Topological matter, Electronics, photonics and device physics

## Abstract

Topological Insulators (TIs) are promising platforms for Quantum Technology due to their topologically protected surface states (TSS). Plasmonic excitations in TIs are especially interesting both as a method of characterisation for TI heterostructures, and as potential routes to couple optical and spin signals in low-loss devices. Since the electrical properties of the TI surface are critical, tuning TI surfaces is a vital step in developing TI structures that can be applied in real world plasmonic devices. Here, we present a study of Bi_2_Se_3_/graphene heterostructures, prepared using a low-cost transfer method that reliably produces mono-layer graphene coatings on Bi_2_Se_3_ flakes. Using both Raman spectroscopy and electron energy loss spectroscopy (EELS), we show that the graphene layer redshifts the energy of the $\pi$ plasmon mode in Bi_2_Se_3_, creating a distinct surface plasmon that differs significantly from the behaviour of a TI-trivial insulator boundary. We demonstrate that this is likely due to band-bending and electron transfer between the TI surface and the graphene layer. Based on these results, we outline how graphene overlayers can be used to create tuneable, stable plasmonic materials based on topological insulators.

## Introduction

Topological Insulators (TIs) are useful platforms for quantum technologies due to the Topological Surface State (TSS), which exhibits phenomena such as spin-momentum locking, and the potential for these states to host exotic entangled particles, such as Majorana bound states^1–3^. Recently, TIs have also attracted attention as platforms for plasmonic devices at UV, optical and THz frequencies^4–6^. This is because the highly tuneable, high conductivity surface states of TIs provide an effective platform for confining Dirac plasmon excitations in two dimensions. Furthermore, since carriers in the TSS are topologically protected against back-scattering, these materials present a route to extremely low-loss plasmon devices^7^. Finally, spin-momentum locking suggests that acoustic plasmon excitations in extremely thin TI layers could provide efficient spin-charge separation, which could integrate plasmonic and spintronics devices^8^.

In order to realise any of these technologies it is necessary to develop methods to tune and protect the surface state^9^. TIs exposed to air typically form a two dimensional electron gas on their surface as a result of adsorbed contaminant layers that build up on the terminating surface^10^. This can also be illustrated by implanting metallic dopants into the surface^11^. Band-bending from these surface impurities pushes the TSS below the Fermi energy. A promising route to tuning and protecting this surface is to coat it with another two dimensional material. In particular, graphene/TI interfaces show gate-tuneability and a strong photo-response in both infra-red (IR) and optical regimes^12–14^.

Currently, graphene/TI devices are usually developed with the graphene as a substrate layer, since high-quality graphene films can be grown via thermal decomposition of SiC or via chemical vapour deposition (CVD) onto SiO_2_^14–16^. Transfer or growth of graphene onto TI films tends to be accompanied by the formation of a few nm thick layer of contaminants or oxides^13^. This is problematic for applications, since plasmonic devices, especially those utilising acoustic excitations coupled across ultra-thin films, require tuneability of both surfaces simultaneously. Here, we have utilised a wet transfer process to encapsulate Bi_2_Se_3_ flakes in graphene single layers. Utilising a low temperature baking process, we were able to achieve a high quality Bi_2_Se_3_/graphene interface with good coverage and minimal contaminants. Such surfaces could be further functionalised by coating with organic dyes, which can be used to tune Rashba coupling in the interface state^17,18^. We then examine the plasmonic behaviour of the interface using low-loss EELS, particularly the interband transitions associated with the $\pi$ and $\pi +\sigma$ transitions. These excitations have been shown to provide a useful measure of changes in surface potential, hybridisation and 2D confinement of the surface states^19^.

## Results

### Sample preparation

**Figure 1:**
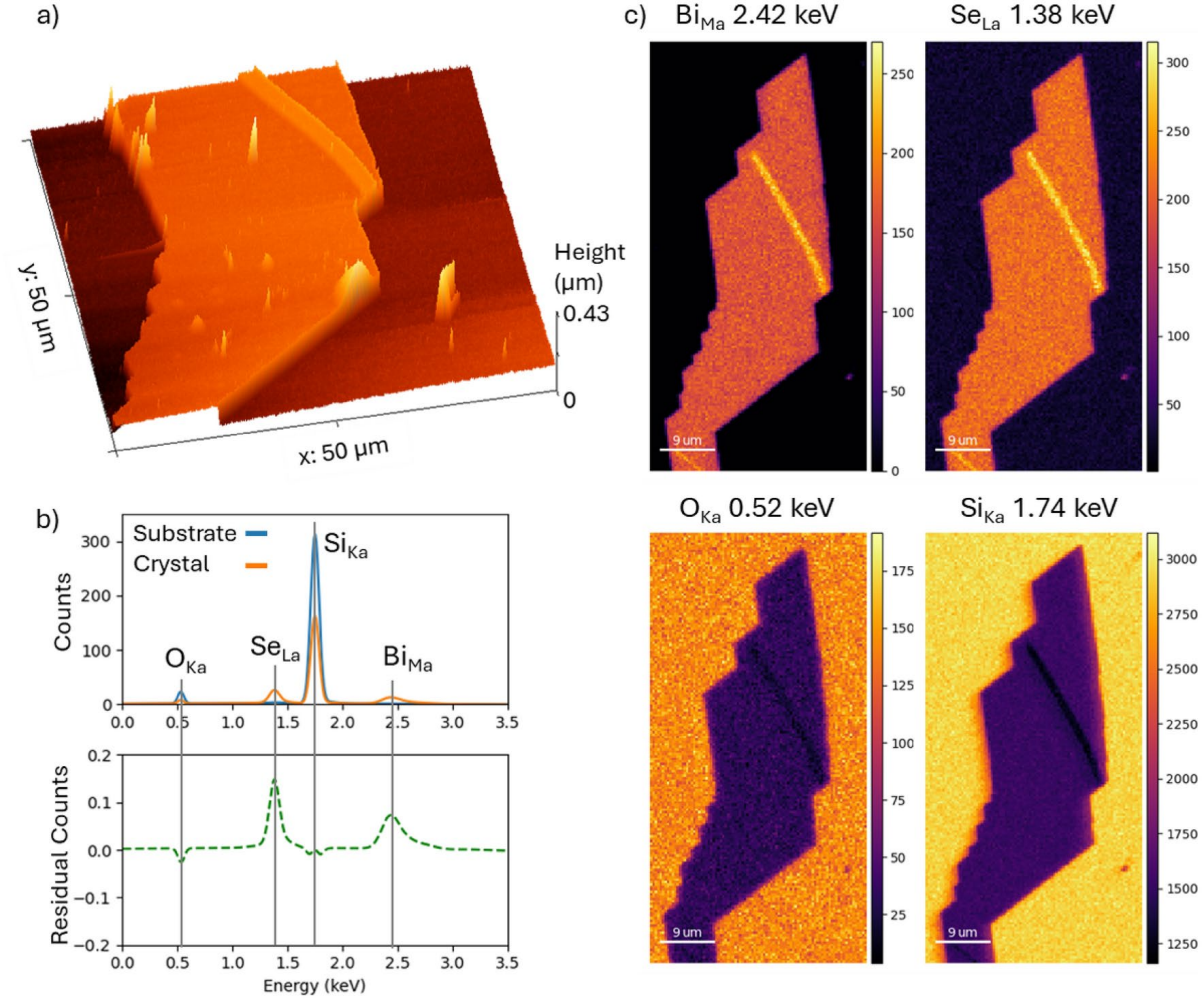
(**a**) AFM relief map of single Bi_2_Se_3_ flake on SiO_2_. (**b**) EDS counts from both substrate and flake. (**c**) EDS map of the flake for relevant elements.

Single crystal flakes of Bi_2_Se_3_ were produced on an SiO_2_ substrate using a vapour transport growth methodology in a sealed ampule with an inert argon atmosphere. The argon had a purity of 99.999%. In order to achieve the desired growth of the crystals a polycrystalline source of Bi_2_Se_3_ pre-prepared from high-purity elements of bismuth (Bi, 99.999%) and selenium (Se, 99.999%) with the melt-growth method was placed inside a quartz ampule along with a silicon substrate. This ampule underwent a series of pumping and flushing cycles with the pure argon before the ampule was left in a depressurized state. Subsequently, the ampule was sealed with an oxy-acetylene torch^20^. The growth of the crystals utilizes a 3-zone horizontal tube furnace capable of reaching temperatures up to 1000 °C, allowing for control of the temperature gradient over the ampule. For the entirety the growth a source temperature of approximately 600 °C^21–23^ was maintained in addition to a substrate temperature of approximately 550 °C and a temperature gradient of 7 °C $\hbox{cm}^{-1}$ over the duration of a 35 hr growth. After growth, the crystals were characterised with Raman spectroscopy, Atomic Force Microscopy (AFM) and Scanning Electron Microscopy (SEM) to verify the correct phase, size, and homogeneity. Stoichiometry of individual flakes was confirmed with EDS mapping and surface topography was assessed using AFM, Fig. 1. Individual flakes contain small numbers of crystallites and nanowire growths. Apart from these defects, flakes have RMS roughness $< 1.25$ nm. A 2:3 ratio of Bi to Se was observed uniformly across flakes, with increased total emission from crystallites and nanowires.

A CVD grown single layer of graphene on a copper foil was obtained from Graphenea. The transfer process of graphene onto the Bi_2_Se_3_ sample followed a wet transfer method. Initially, a layer of polymethyl methacrylate (PMMA) was spin-coated onto the graphene/copper foil stack at 7700 RPM. This PMMA layer served as a protective buffer to protect the graphene and minimize crack formation during the transfer process. Subsequently, the PMMA/graphene/copper film stack was floated on a copper etchant solution consisting of ammonium persulfate (APS) (0.06 g/mL in H_2_O) for 12 h to dissolve the copper layer. Following this step, the PMMA/graphene stack underwent rinsing by floating it on two successive baths of ultra-pure de-ionised (DI) water for up to 1 h. The PMMA/graphene film was carefully transferred onto the Bi_2_Se_3_ sample by lifting it from beneath the water surface. The PMMA/graphene/Bi_2_Se_3_ stack was then subjected to a 4-hour baking process on a hot plate at 87 °C to enhance the adhesion of graphene to the substrate and drive off residual water. Subsequently, the PMMA/graphene/Bi_2_Se_3_ stack underwent an overnight immersion in acetone with magnetic stirring to dissolve the PMMA layer. Finally, the sample was rinsed with isopropyl alcohol (IPA) and DI water.

### Raman characterisation

**Figure 2:**
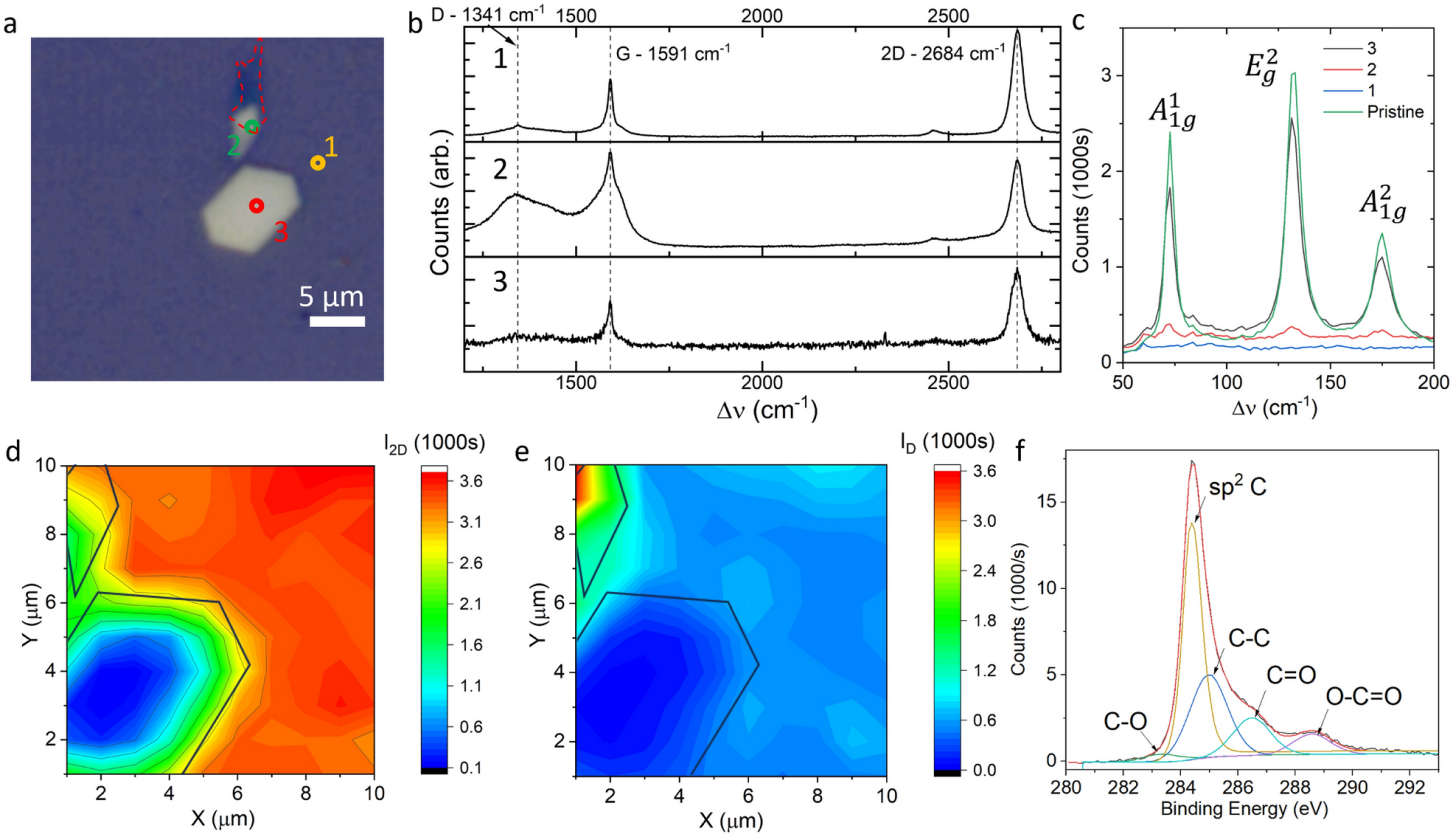
(**a**) Optical microscope image of the single crystal flake at 50× magnification. The red outline shows a tear in the graphene layer which we will use to compare intact and damaged coverage (**b**) Raman spectra in the three regions marked in a, with the graphene modes marked. (**c**) Raman spectra for the Bi_2_Se_3_ phonons in the three regions marked in a. (**d**) Intensity of the 2D peak across the map area with the boundary of the flake marked. (**e**) Intensity of the D peak across the map area. (**f**) XPS C1s emission from graphene transferred to a reference substrate.

A single crystal of Bi_2_Se_3_ which had a mirror flat surface and dimensions of 10 × 7 μm, was identified for Raman spectroscopy (Fig. 2a). The flake was accompanied by a small satellite flake with a break in the graphene coverage, visible as a darker region above the flake, which provides a useful point of comparison between full coverage and damaged coverage. The 2D mode, which involves two D band phonons with opposite momenta, is stronger in single layer graphene, while the G mode, which is a single phonon process, is present in single-layer and multi-layer graphene. Thus, the intensity ratio $I_{2D}/I_G$ can tell us whether we have single layer graphene. On the substrate and flake, positions 1 and 3 in Fig. 2a, the ratio is $1.8 \pm 0.1$ and the narrow 2D peak has a frequency $2684 \,\text{cm}^{-1}$ which indicates single layer graphene, Fig. 2b (1,3)^24,25^. Around the defect at position 2, the $I_{2D}/I_G$ ratio is 0.85. The D peak depends on defect scattering and is therefore indicative of the number and distance between defects in the graphene film. At position 2, the broad D peak and low $I_{2D}/I_G$ indicates an edge with significant amorphisation or folding, Fig. 2b(2)^26,27^. The Raman peaks associated with the Bi_2_Se_3_ phonons show a $A_{1g}^1/A_{1g}^2$ ratio of $1.5 \pm 0.1$ which indicates bulk behaviour, Fig. 2c^28^. The Raman spectrum of the covered Bi_2_Se_3_ is identical to pristine flakes prior to transfer, with only slightly reduced intensity, indicating the Bi_2_Se_3_ has not been affected by the transfer process.

The graphene Raman signal was mapped across the flake and surrounding substrate, Fig. 2d,e. The G, D and 2D peaks are all suppressed over the Bi_2_Se_3_ flake without any change in the peak ratio or peak positions. This indicates that the Raman suppression is not a result of damage to the graphene layer, strain or other changes to the layer structure^29^. Instead, this is consistent with a charge transfer process, affecting exciton lifetime at the heterostructure interface. Such transfer processes have been observed in graphene/Bi_2_Se_3_ heterostructures, even with an interfacial BiO_2_ layer^13^. Without this oxide layer, electron-hole separation due to interfacial band-bending is expected to have significant effects on exciton dynamics, since there is a Fermi level mismatch of 0.8 eV at the interface^30^. The high D peak intensity and drop in $I_{2D}/I_G$ ratio is localised around the tear in the graphene layer and is weak everywhere else. This indicates that while the graphene layer is not defect free, defect concentration is low after the transfer process and the transferred graphene is consistently single layer. Graphene layers transferred to reference SiO_2_ substrates were analysed using X-ray Photoemission Spectroscopy (XPS), Fig. 2f. XPS showed complete removal of the PMMA layer with only trace amounts of $\hbox {PMMA}^G$ 2D residuals on the upper surface, which are insoluable to acetone. These residuals are far less than a monolayer based on comparison to literature, and exist on the upper surface, not at the interface^31^. Raman and XPS spectra do not show evidence of APS or Acetone residue. However, it should be noted that trace amounts of solvent may be localised to specific flakes. Details of the transfer process have been discussed in previously published work^32^.

**Figure 3:**
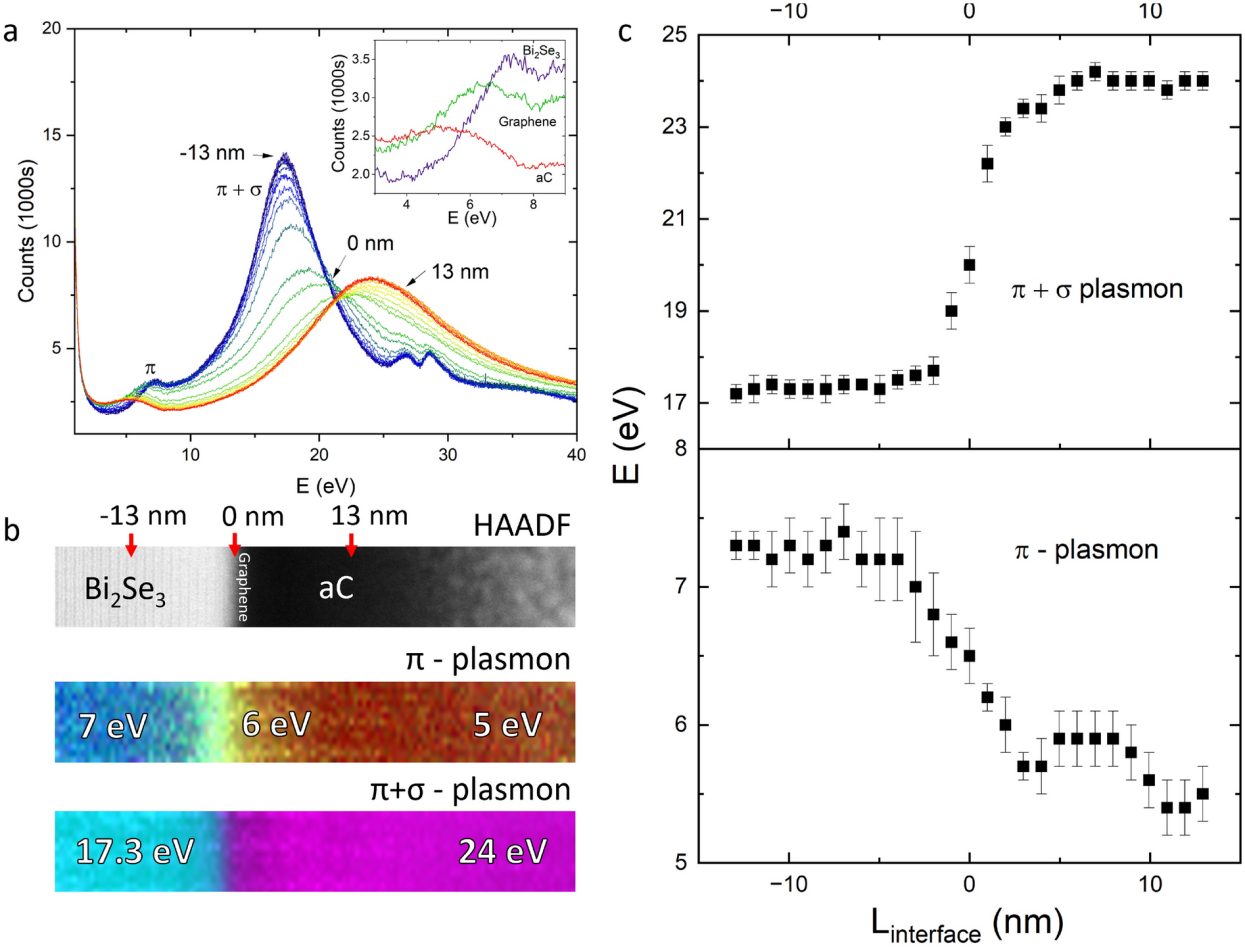
(**a**) Low loss EELS spectra obtained as a function of distance from the Bi_2_Se_3_/graphene/aC interface. The scanned region spans 60 nm with the positive direction running left to right in the images in (**b**). Blue lines indicate spectra from the bulk Bi_2_Se_3_ up to a position of − 13 nm, while red indicates spectra from the bulk aC up to a position of +13 nm. Green indicates the interface. The inset shows a zoom in of the $\pi$ plasmon mode. (**b**) Spectral maps and HAADF collected from the spectral image. For the $\pi$ plasmon, the red channel is the integrated spectral density between 4.5 and 5.5 eV, the green channel is between 5.5 and 6.5 eV and the blue channel is between 6.9 and 7.9 eV. For the $\pi + \sigma$ plasmon, the cyan channel is between 17 and 18 eV and the mageneta channel is between 23.5 and 24.5 eV. (**c**) The energy of the plasmon peaks plotted as a function of position between −13 nm and +13 nm with 0 centred on the approximate position of the graphene layer.

### EELS analysis

Electron energy loss spectroscopy (EELS) was carried out using a JEOL ARM200cF with a Gatan 965 Quantum ER spectrometer. Scanning transmission electron microscopy (STEM) imaging through a sample cross section was performed at 200 kV with a 0.1 nm probe. The low-loss spectrum was acquired across a 60x9 nm region intersecting the Bi_2_Se_3_/graphene interface over the energy range 0–40 eV, Fig. 3a. A protective amorphous carbon (aC) layer was added during focused ion beam (FIB) extraction. Moving from the Bi_2_Se_3_ (blue lines) to the amorphous carbon (red lines) in 0.5 nm steps, we observe the evolution of the plasmon spectrum approaching the interface. Within the bulk Bi_2_Se_3_, 13 nm from the interface, the EELS spectrum closely matches that observed in other EELS studies of crystalline Bi_2_Se_3_^19^. The $\pi$ plasmon peak at 7 eV is due to excitation of $\pi$ electrons in the material bulk, while the $\pi + \sigma$ feature at 17.3 eV involves plasma excitations of both $\pi$ and $\sigma$ electrons. The two small features at 26.7 eV and 28.5 eV are attributed to the $O_{4,5}$ core loss EELS edges of Bi. In amorphous carbon, the bulk plasmon peak is located at 24 eV and the broad peak at 5 eV is associated with a $\pi \rightarrow \pi ^*$ transition^33^.

In freestanding graphene monolayers, the $\pi$ plasmon mode is expected to be at 4.7 eV^34^. However, when held on a substrate, increased screening causes the $\pi$ plasmon energy to shift to between 5.9 eV and 6.2 eV. Graphite, by comparison, shows a bulk $\pi$ plasmon mode between 6.5 eV and 7 eV^35^. The graphene $\pi + \sigma$ mode is expected at 14.7 eV, and is very weak in freestanding monolayers^36^. However, on substrates such as SiC, even double layer graphene shows a blueshifted and amplified $\pi + \sigma$ peak at 19 eV^35^. Doping also has a significant effect on this peak, with metal doped graphene films showing a $\pi + \sigma$ excitation at 20 eV^37^. Therefore, we can infer from the literature that the energy of the $\pi + \sigma$ plasmon is very dependent on substrate and doping and is therefore difficult to distinguish in this heterostructure.

Measuring the normalised amplitude of the EELS response in these regions of interest provides intensity maps of the different excitations across the interface, Fig. 3b. Aligning this with the high-angle annular dark field (HAADF) image allows us to observe where different plasmons are localised. The 7 eV bulk $\pi$ plasmon and 5 eV $\pi \rightarrow \pi ^*$ excitations are localised to the Bi_2_Se_3_ and aC layers respectively. At the interface, the $\pi$ plasmon mode is linearly red-shifted to 6 eV. Integrating the spectral density between 5.5 and 6.5 eV shows that the redshift occurs linearly over a region ± 5 nm from the interface, Fig. 3c (bottom). In comparison, the $\pi + \sigma$ plasmon blueshift occurs only within 1 nm of the interface, 3c (top). The energy of these two excitations can be plotted as a function of distance from the interface to show this change. The peak positions are determined by fitting a Voigt function with a zero-loss correction. The method is detailed in the discussion section.

**Figure 4:**
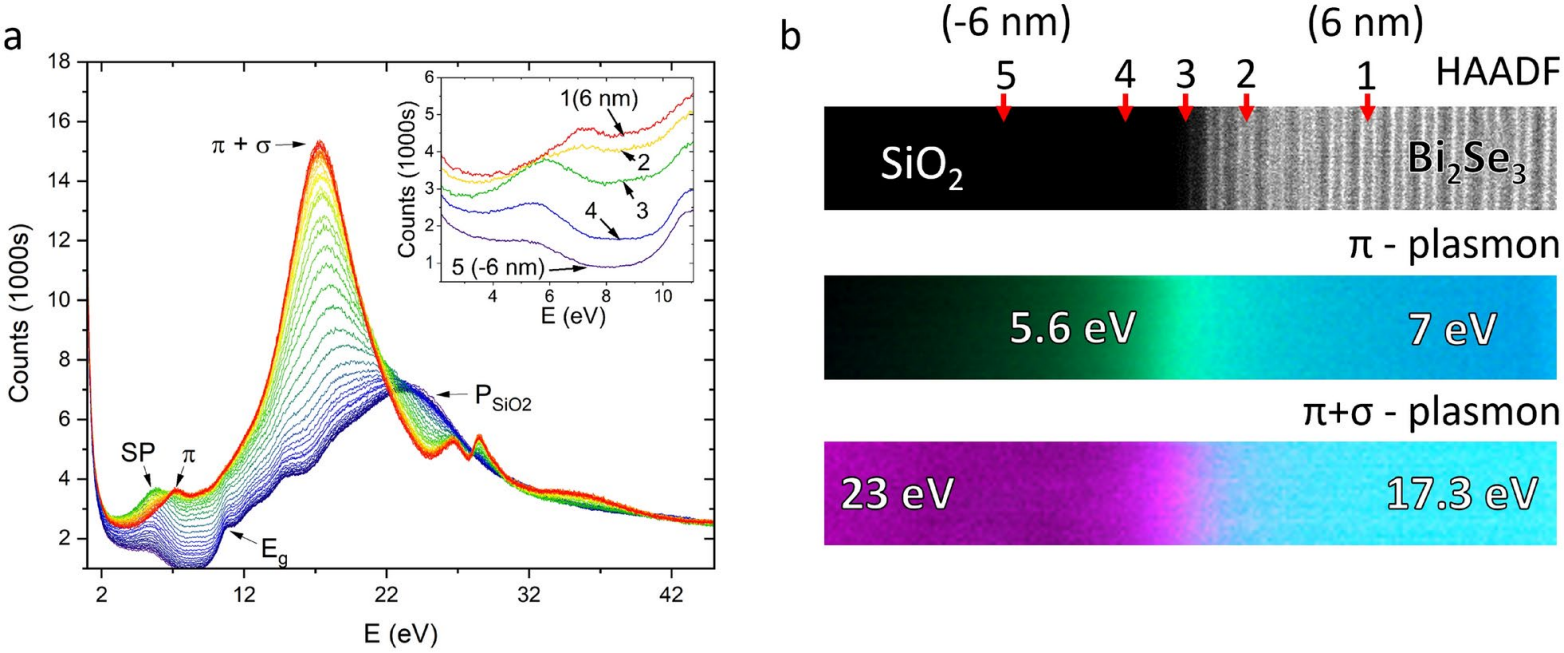
(**a**) Low-loss EELS spectra recorded across the Bi_2_Se_3_/SiO_2_ interface ranging from the bulk Bi_2_Se_3_ at 6 nm (red) to the bulk SiO_2_ at − 6 nm (blue) with 0 nm (green) corresponding to the interface. The inset shows a highlight of the $\pi$ plasmon region with labels 1 to 5 corresponding to the locations marked on the HAADF image. Spectra are offset for clarity. (**b**) HAADF and spectral maps of the Bi_2_Se_3_/SiO_2_ interface. For the $\pi$ plasmon, the blue channel corresponds to 6.9–7.9 eV. The green channel corresponds to 5–6 eV. For the $\pi + \sigma$ plasmon, the cyan channel corresponds to 17–18 eV and the magneta channel corresponds to 22.5–23.5 eV.

An interesting comparison can be made to the interface between the Bi_2_Se_3_ and the SiO_2_ substrate. Here, the interface is scanned over a region of 12 × 3.5 nm. The bulk Bi_2_Se_3_ spectrum in Fig. 4a is identical to that recorded in Fig. 3a while the spectrum of the substrate closely matches literature examples of amorphous SiO_2_, Fig. 4a^38^. A bulk plasmon peak is expected in SiO_2_ at 23 eV with inter-band transitions at 18.2, 14.3, 12.0 and 10.6 eV. The band edge ($E_g$) is observed at 9.4 eV. The feature at 5–6 eV is best understood as an aloof excitation of the surface plasmon (SP) mode. In some examples of amorphous SiO_2_ a second band edge is observed at 5.5 eV due to impurities, however the feature observed here does not have the step characteristics of a band edge. As we approach the interface, we observe this mode increase in intensity and shift to 5.6 eV, persisting into the first layer of the Bi_2_Se_3_. One quintuple layer below the Bi_2_Se_3_ surface, this feature has vanished, and the bulk $\pi$ plasmon mode at 7 eV has appeared. A comparison of the $\pi$ plasmon mode in different regions is given in Fig. 4a (inset), with labels 1 to 5 corresponding to the marked positions in the film indicated in Fig. 4b. In the interfacial region, it is evident there are two excitations, one localised at the interface and one present in the bulk, whose intensities are dependent on position. Since the 5.5–5.6 eV mode is strongest at the interface and is present in the bandgap, we assign it to the surface plasmon (SP) mode observed by Liou^19^, excited in aloof mode by the beam in the insulating SiO_2_ layer.

## Discussion

**Figure 5:**
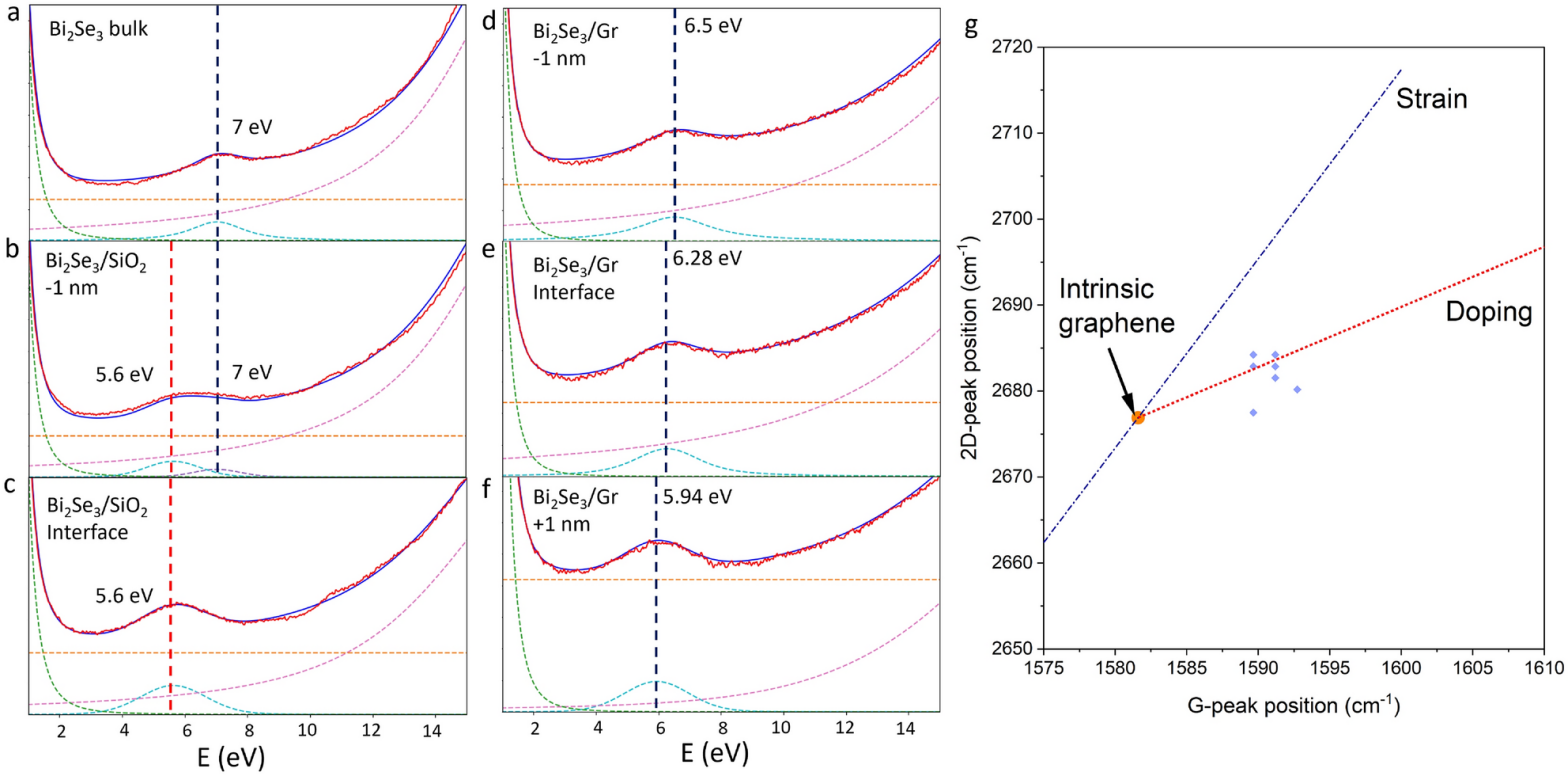
The spectra were fit with Voigt functions to extract the peak positions. The total fit is marked in red and original data in blue. Seperate peak fits are plotted as dotted lines with the $\pi$ plasmon and SP modes marked. A power law was used to model the edge of the zero-loss peak and a linear offset was applied. (**a**–**c**). Fits of the $\pi$ plasmon and SP mode in the Bi_2_Se_3_ bulk (**a**), 1 nm from the Bi_2_Se_3_/SiO_2_ interface showing the two peak best fit (**b**) and at the interface (**c**), in which the band edge is just visible at 10 eV. (**d**–**f**). Fits of the $\pi$ plasmon mode 1 nm into the Bi_2_Se_3_ (**d**) at the Bi_2_Se_3_/graphene interface (**e**), and 1 nm into the graphene/*aC* (**f**) showing the shift of the $\pi$ plasmon energy. (**g**) Plot of G and 2D peak positions measured via Raman on the Bi_2_Se_3_/graphene flake with the strain (blue) and doping (red) vectors marked. The blue diamonds are the peak positions measured over the flake.

A comparison of the EELS spectra at the two Bi_2_Se_3_ interfaces shows very different behaviour for a TI/insulator and TI/graphene interface. The TI/insulator interface shows very similar trends to those observed in TI/vacuum interfaces, owing to the fact that vacuum is, like SiO_2_ a trivial insulator^19,39^. The SP mode in the Bi_2_Se_3_/SiO_2_ interface at 5.6 eV and that observed at the vacuum interface, 5.5 eV, almost match in energy. This is an important point to emphasise since SiO_2_ is a CMOS compatible material. The small difference can be explained by the presence of amorphous SiO_2_, which is likely to contain small numbers of surface defects^38^. The fact that the SP mode is excited well into the SiO_2_ despite existing in the band gap, clearly identifies it as a surface mode. Moving between the positions 1 to 5 identified in Fig. 4a (inset) and b, the relative amplitude of the bulk and SP mode change. This can be more easily observed by fitting each peak with Voigt functions as shown in Fig. 5a–c. One quintuple layer (roughly 1 nm) below the surface, marked as spectrum 2 in Fig. 4a (inset) and b, a two peak model is required to fit the $\pi$ plasmon mode. This is in contrast to the Bi_2_Se_3_/graphene interface. As we approach this surface in Fig. 3, the $\pi$ plasmon peak is continuously red-shifted over a 10 nm interfacial region. Across the interface, this feature is best fit as a single peak, as can be seen in Fig. 5d–f. This length scale closely matches the depth to which band bending is predicted^14^ and measured^16^ to extend in Bi_2_Se_3_ deposited on graphene substrates.

Unlike the Bi_2_Se_3_/SiO_2_ interface, the Bi_2_Se_3_/graphene interface is always best fit with a single peak. The expected 4 eV peak for freestanding monolayer graphene is not present, however, the change in local screening, interfacial potential and carrier density in an interface versus a freestanding layer is expected to shift the energy of the $\pi$ electron excitations in graphene close to 6 eV, which is the energy of the $\pi$ plasmon measured at the Bi_2_Se_3_/graphene interface, Fig. 5e^35^. The doping of the graphene is directly evidenced by the Raman map. By comparing the G and 2D peak positions, we can use a vector decomposition technique to determine whether the graphene layer is strained or doped^40^. On the Bi_2_Se_3_ flake shown in Fig. 2, we see our graphene sits on the zero strain, hole-doped line, with a doping concentration of $3.1 \pm 0.1 \times 10^{11} \,\text{cm}^{-2}$, Fig. 5g. The sputtered aC will affect this doping, as evidenced by the use of aC/multi-layer-graphene heterostructures as p-n junctions for solar cells, so it is not possible to precisely calculate the expected shift of the graphene $\pi$ plasmon, but the measured energy of 6 eV is reasonable given literature^41,42^. Exchanging the aC layer for different overlayers could be used to tune this hybrid interface further to achieve different optical plasmonic effects and shift the energy of the $\pi$ plasmon excitation.

## Conclusion

From the shift in the surface $\pi$ plasmon mode at the Bi_2_Se_3_/graphene interface, we can infer that graphene overlayers provide a simple route to protect and tune the plasmonic properties of these interfaces. Adding different dopant layers to the monolayer graphene will further modify the interface through band-bending or electron doping, without directly contaminating the TI surface or introducing time-reversal symmetry breaking impurities that would gap the Dirac surface state. The relative ease with which these materials can be prepared, without introducing thick *BiO* or other contaminants into the surface makes this a viable method to tune the properties of plasmonic devices that incorporate topological insulators, opening up a route forward for engineering topological plasmonic devices that can couple optical and spin signals, efficiently transform THz signals or act as plasmonic sensors.

## Methods

### FIB lamella extraction

Cross-sections were extracted for STEM using focused ion beam (FIB) techniques. The bulk material was sputter coated with around 15 nm of amorphous carbon (aC) to protect the graphene layer. After deposition of a protective Pt cap, a lamella of size $20\times 3\, \upmu \text{m}$ was extracted using a 30 kV Xe ion beam of currents up to 6.7 nA on a Helios Xe Plasma FIB instrument. The extracted lamella was welded to a copper TEM holder with Pt. The lamella was thinned until electron transparent at − 175 °C using a NOVA 200 Ga FIB. Thinning was performed in three main steps with a thick unmilled region left on either side of the thinned area. Firstly, a large region of material was removed by a 30 kV 93 pA beam at tilt angles of $\pm 1.5^{\circ }$ from both the front band back faces. Secondly, the tilt angle was reduced to $\pm 1.2^{\circ }$ and cleaning cross sections used with the same beam conditions to further thin the lamella until electron transparent. Finally, the cross-section was polished with a 5 kV 47 pA beam.

### Raman method

The quality of the transferred graphene was evaluated optically using a microscope and Raman spectroscopy. Raman spectroscopy analysis was performed using the HORIBA LabRAM HR Evolution Raman spectrometer with a laser wavelength of 532 nm (corresponding to an excitation energy $E_L = \hbar wL = 2.33 \,\text {eV}$). The experimental setup included an optical fiber and a 100X objective lens with a numerical aperture (NA) of 0.8, resulting in a laser spot size of 2 μm. To ensure that the laser power remained below 2 mW, a neutral density (ND) filter was employed. The Raman peak positions were calibrated using the silicon peak at 520.7 cm$^{-1}$ as a reference.

## Data Availability

Data supporting the results presented in this work are available from the corresponding author on reasonable request.
